# Effects of HD-tDCS combined with Bosu ball training on static and dynamic postural stability among individuals with chronic ankle instability

**DOI:** 10.3389/fspor.2025.1618683

**Published:** 2025-06-25

**Authors:** Yubin Ge, He Gao, Xueke Huang, Xin Luo, Yanhao Liu, Dongmei Wang, Peixin Shen, Liang Guo, Qipeng Song

**Affiliations:** ^1^ College of Sports and Health, Shandong Sport University, Jinan, China; ^2^ Physiotherapy Programme, Centre for Healthy Ageing and Wellness, Faculty of Health Sciences, Universiti Kebangsaan Malaysia, Kuala Lumpur, Malaysia; ^3^ Sport Science School, Beijing Sport University, Beijing, China; ^4^ School of Physical Education & Sports Science, South China Normal University, Guangzhou, China

**Keywords:** ankle sprains, neuromuscular control, non-invasive brain stimulation, postural control, unstable surface training

## Abstract

**Purpose:**

Chronic ankle instability (CAI) is characterized by a combination of peripheral dysfunction and maladaptive neuroplasticity in central nervous system, leading to persistent postural stability deficits. This study aims to investigate the effects of high-definition transcranial direct current stimulation (HD-tDCS) combined with Bosu ball training (BBT) on the static and dynamic postural stability in individuals with CAI.

**Methods:**

A total of forty participants were randomized to receive either HD-tDCS + BBT (*n* = 20) or BBT (*n* = 20) interventions, delivered over six weeks with three 20-minute sessions per week. Static and dynamic postural stability was assessed pre- and post-intervention via single-leg stance and drop landing tests, with kinetic data captured by a force platform (1,000 Hz). Data were analyzed using two-way mixed-design ANOVA.

**Results:**

Significant group-by-time interactions were detected in the center of pressure-root mean square (CoP_RMS) during single-leg stance (*p* = 0.036, *η*^2^*ₚ* = 0.134) and the time to stabilization (TTS) during drop landing (*p* = 0.007, *η*^2^*ₚ* = 0.209) in the mediolateral (ML) direction. *Post hoc* comparisons showed that the both of them were decreased after intervention, and greater decreases were observed by the intervention of HD-tDCS + BBT compared to BBT. And, a significant time main effect was observed in the CoP_RMS (*p* < 0.001, *η*^2^*ₚ* = 0.382) and the TTS (*p* = 0.005, *η*^2^*ₚ* = 0.224) in the anteroposterior direction, they both decreased after HD-tDCS + BBT and BBT interventions.

**Conclusions:**

Both BBT alone and the combined HD-tDCS + BBT interventions enhanced static and dynamic postural stability in individuals with CAI, while the combined HD-tDCS + BBT intervention demonstrated significantly greater efficacy in improving postural stability in the ML direction compared to BBT alone.

## 1 Introduction

Lateral ankle sprains (LAS) represent a prevalent category among musculoskeletal injuries, constituting 10%–30% of all reported cases (1, 2). It is estimated a daily incidence exceeding 25,000 ankle sprain cases in the United States alone (3). Approximately 46% of LAS progressing to chronic ankle instability (CAI) (4), characterized by persistent symptoms such as recurrent ankle sprain, “giving way”, pain, and weakness, which may lead to long-term neuromuscular damage and an increased risk of osteoarthritis (5–7). It is estimated that approximately 2 million acute ankle sprains occur annually in the United States (8), resulting in medical costs of about $6.2 billion (9).

Individuals with CAI exhibit disruptions in sensory-motor integration, a critical process for maintaining postural stability. This impairment arises from the damage to mechanoreceptors and afferent fibers in the ankle joint due to recurrent sprains (10), and the maladaptive neuroplastic changes in sensorimotor cortical regions, particularly the primary motor cortex (M1) and primary somatosensory cortex (S1) (11). Postural stability relies on the integration of somatosensory inputs, central nervous system (CNS) processing, and motor outputs that coordinate muscle activity to regulate joint loading and balance (12). In CAI, mechanoreceptor dysfunction compromises sensory input from the ankle, while cortical reorganization in M1 and S1 alters neuromuscular control pathways (13). These combined deficits impair the CNS's capacity to modulate joint mechanics and muscle activation patterns, perpetuating postural instability and functional limitations.

Individuals with CAI demonstrate deficits in both static and dynamic postural stability, which are critical for injury prevention and functional performance. Static postural stability is commonly assessed via root mean square (RMS) of center of pressure (CoP) displacement during single-leg stance (14), which effectively predicts lower-limb injury risk and monitors rehabilitation progress (15). Compared to healthy controls, individuals with CAI exhibit greater CoP_RMS, particularly under open-eye conditions (16). Dynamic postural stability, usually evaluated through time to stabilization (TTS) during drop-landing tasks, quantifies the ability to maintain balance during high-demand activities (17). Individuals with CAI demonstrate prolonged TTS, indicating delayed neuromuscular adjustments and reduced dynamic control (18).

Conventional CAI interventions, such as sensory-targeted training (19), cryotherapy (20), ankle joint mobilization (21), and plantar massage (12), primarily target peripheral deficits (e.g., tactile sensation, proprioception, muscle strength). However, these approaches often yield limited efficacy, with persistent instability or recurrent injury in many cases (22, 23). This may reflect inadequate consideration of maladaptive CNS neuroplasticity, now recognized as a key contributor to CAI-related postural deficits (11, 15). Previous studies demonstrates reconceptualizing CAI as a global sensorimotor integration disorder rather than a localized peripheral injury, with neuroplastic maladaptations observed in cortical regions associated with postural stability (24, 25). Multimodal strategies integrating CNS interventions with peripheral therapies are needed.

Emerging evidence supports transcranial direct current stimulation (tDCS) as a promising CNS rehabilitation strategy for CAI, and a more advanced variant, high-definition tDCS (HD-tDCS), employs compact circular electrode arrays to modulate cortical excitability, enhance neuroplasticity, and improve postural stability with superior spatial specificity and prolonged physiological effects (26–28). Preliminary studies in healthy adults demonstrate that HD-tDCS enhances postural stability (29), suggesting its potential to address CNS-mediated deficits in CAI. Critical to the efficacy of HD-tDCS is the pairing of stimulation with task-specific motor training, as concurrent activation of sensorimotor networks during stimulation amplifies motor learning and skill acquisition (30). For CAI rehabilitation, progressive balance exercises—gradually increasing in complexity—may synergize with HD-tDCS by challenging sensorimotor adaptability and refining motor planning strategies, thereby enhancing postural control (31). For instance, Bosu ball training (BBT)—which creates an unstable surface environment—could serve as an effective paired task, as it demands continuous proprioceptive integration and reactive postural adjustments (30–33).

As mentioned above, postural stability plays a critical role in individuals with CAI, where postural instability serves as a key contributor to recurrent ankle sprains. While tDCS has demonstrated efficacy in improving postural stability in CAI populations, existing studies have separately investigated static and dynamic postural stability (29, 34–36). To our knowledge, no studies have investigated the effects of HD-tDCS on both static and dynamic postural stability concurrently, particularly when combined with task-specific motor training such as BBT. This study aims to investigate whether HD-tDCS paired with BBT enhances postural stability in individuals with CAI compared to BBT alone, hypothesizing that (1) Both the HD-tDCS combined with BBT intervention and the BBT alone could significantly improve static and dynamic postural stability in individuals with CAI, represented by CoP_RMS metric during single leg stance, and TTS during drop landing; (2) the combined HD-tDCS + BBT intervention demonstrates superior improvement compared to BBT alone.

## 2 Materials and methods

### 2.1 Sample size estimation

An *a priori* power analysis (G*Power 3.1) indicated that 26 participants were required to achieve 0.95 statistical power at *α* = 0.05, based on a previous study's group-by-time interaction effect size (*η*^2^*_p_* = 0.122 equals to effects size *f* = 0.372) for CoP_RMS during single-leg stance in individuals with CAI undergoing neuromuscular electrical stimulation (pre: 8.13 ± 1.07 mm vs. post: 6.60 ± 1.14 mm) (37).

### 2.2 Participants

Participants were recruited from August to October 2024 via university e-newsletters, posters, and direct emails. Seventy-five individuals were screened for eligibility using International Ankle Consortium guidelines and additional criteria (38), with 40 providing informed consent. Inclusion criteria were: (1) history of ≥1 ankle sprain >1 year prior with acute symptoms (pain, swelling, activity limitation >1 day); (2) age 18–25 years without athletic specialization; (3) ≥ 2 episodes of ankle instability/"giving way” in the past 6 months; (4) persistent instability/functional impairment; and (5) Cumberland Ankle Instability Tool score <24 (39). Exclusion criteria included lower-limb fractures/surgeries, acute injury within 3 months, bilateral CAI, or neurological disorders impairing motor control (e.g., cerebellar disorders, stroke) (33). The study was approved by the Shandong Sport University Ethics Committee (No. 2023036) and adhered to the Declaration of Helsinki.

### 2.3 Protocol

This single-blind RCT employed a computer-generated random sequence to allocate 40 participants (1:1) into two interventions: (1) HD-tDCS + BBT and (2) BBT (sham HD-tDCS + BBT). Both interventions underwent six weeks of intervention (3 sessions/week, 20 min/session), with HD-tDCS/sham and BBT administered concurrently. The protocol comprised: 10-minute warm-up, four targeted exercises (30-second each, 30-second rest intervals after each exercise, cycle repeated five times), totaling 20-minute of exercise, followed by 10-minute cooldown. Static and dynamic postural stability were assessed pre- and post-intervention, with test sequences randomized via computer-generation to minimize order effects.

### 2.4 High-definition transcranial direct current stimulation

**Figure 1:**
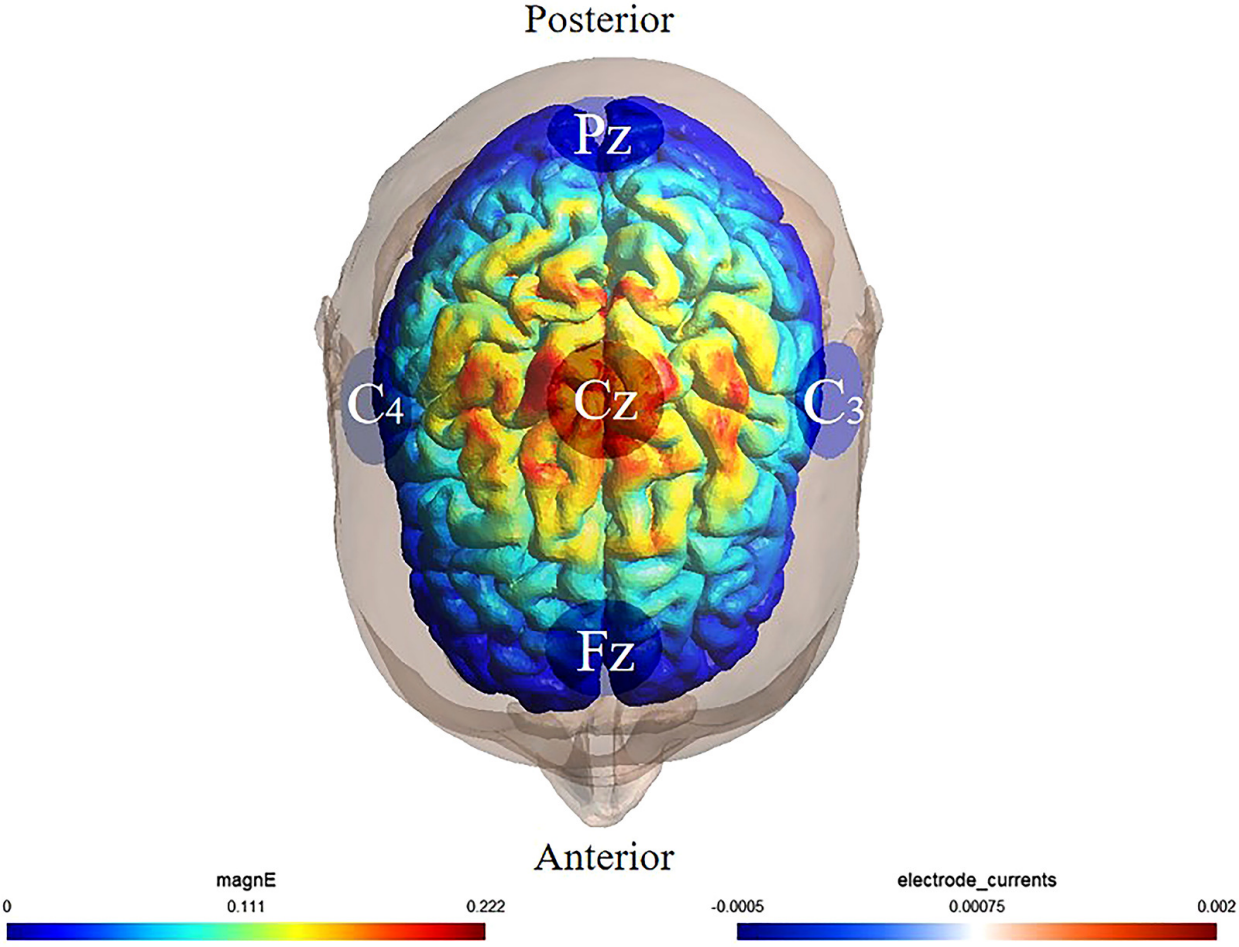
Illustration of HD-tDCS electrode placement. The anode was placed over Cz of the 10/20 EEG template; the four cathodes were placed over Fz, C3, Pz, and C4. Warmer and cooler colors reflect the larger and smaller modeled electric field normal component, respectively.

HD-tDCS was delivered via a StarStim8 device (Neuroelectrics, Spain) using a 10/20 EEG-compliant montage of five 5-mm electrodes: one anode (Cz) and four cathodes (Fz, Pz, C3, C4) (40) (Figure 1). Active stimulation applied 2 mA to the anode, with return current distributed across cathodes. The protocol included a 30-second ramp-up to 2 mA, 19-minute at 2 mA, and a 30-second ramp-down. Sham stimulation mirrored this timing but delivered subthreshold currents (<0.1 mA) during the 19-minute phase to preserve blinding (41). Neuro-modeling confirmed focal targeting of sensorimotor cortices (M1/S1) corresponding to foot-ankle representations (42).

### 2.5 Bosu ball training

**Figure 2:**
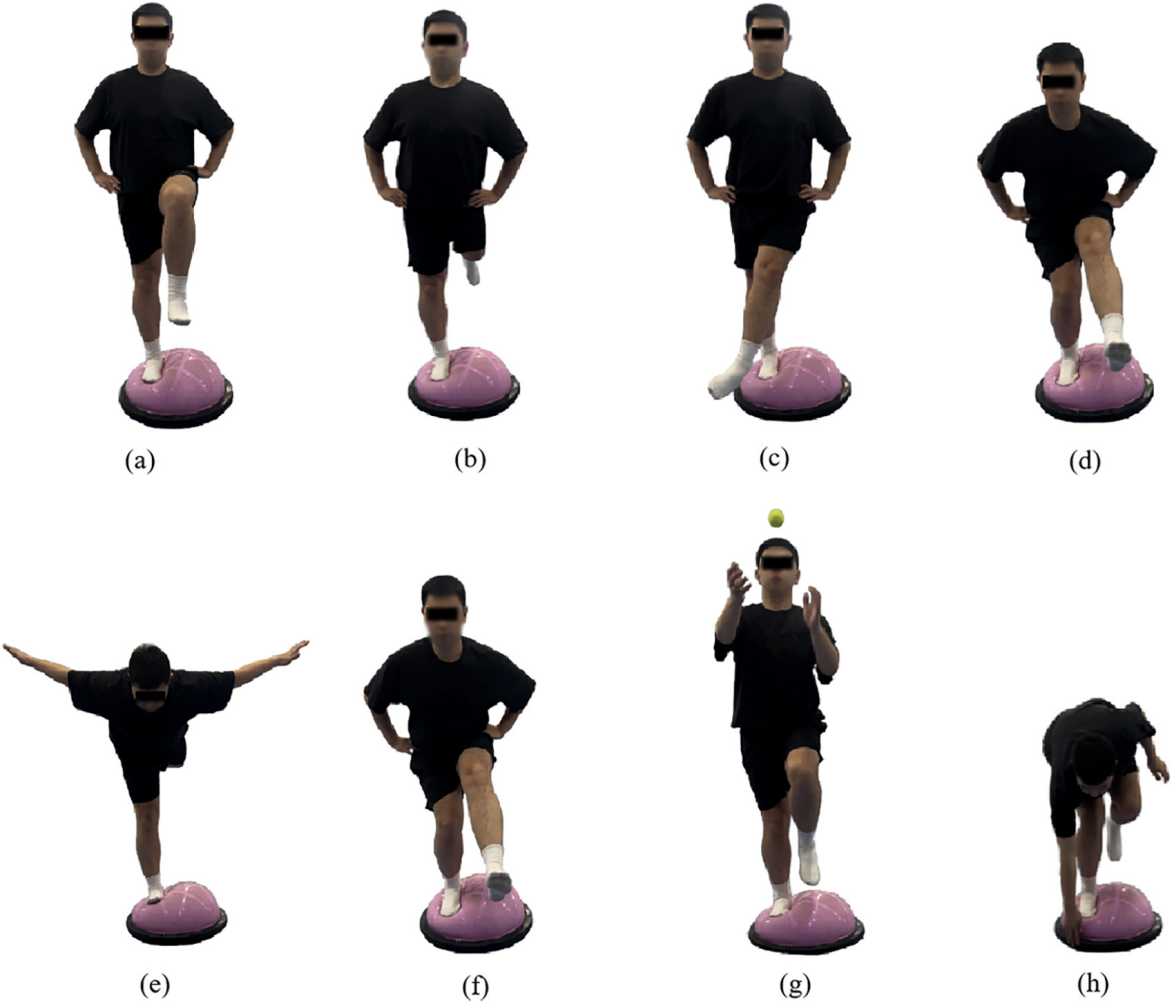
Illustrations of the bosu ball training movements **(a)** single-leg stance, **(b)** single-leg stance with swing forward-backward, **(c)** single-leg stance with swing medial-lateral, **(d)** single-leg squat, **(e)** swallow balanced stance, **(f)** single-legged squat and take-ups, **(g)** catching a ball while single-leg stance, and **(h)** bending over to touch the edge while single-leg stance.

Under the guidance of certified instructors, participants performed Bosu ball training following a progressively intensified program. The intervention involved standing on the soft surface of the Bosu ball with the affected limb positioned superiorly and the unaffected limb adjacent. During weeks 1–2, the training progression consisted of: single-leg stance maintenance; single-leg stance with lower extremity anteroposterior swing (30°–45°); single-leg stance with lower extremity mediolateral swing (20°–30°); and single-leg squats. During weeks 3–4, the training progression consisted of: swallow balance positions; single-leg stance with anteroposterior swing (45°–60°); single-leg stance with lower extremity mediolateral swing(30°–45°); and dynamic single-leg squat take-ups. During weeks 5–6, the training progression consisted of: single-leg stance with ball-catching; single-leg stance with lower extremity anteroposterior mediolateral swing(45°–60°); single-leg stance with lower extremity mediolateral swing (30°–45°); and functional reaching tasks involving forward trunk flexion to touch edge of Bosu ball while maintaining single-leg stability(Figure 2).

### 2.6 Static postural stability test

Participants completed the single leg stance task to assess static postural stability. After reviewing procedures, warming up, and practicing (≥3 trials), they stood on their affected leg atop a force platform (AMTI, Watertown, MA, USA), hands on hips, gaze fixed forward. The unaffected leg was raised to calf level, with the affected foot maintaining full contact for 30 s. Trials were discarded and repeated if: (1) limbs made contact, (2) hands moved from hips, or (3) trunk/hip deviation exceeded 30. Three valid trials were averaged for analysis, with ≥1-minute rest between attempts to minimize fatigue.

### 2.7 Dynamic postural stability test

**Figure 3:**
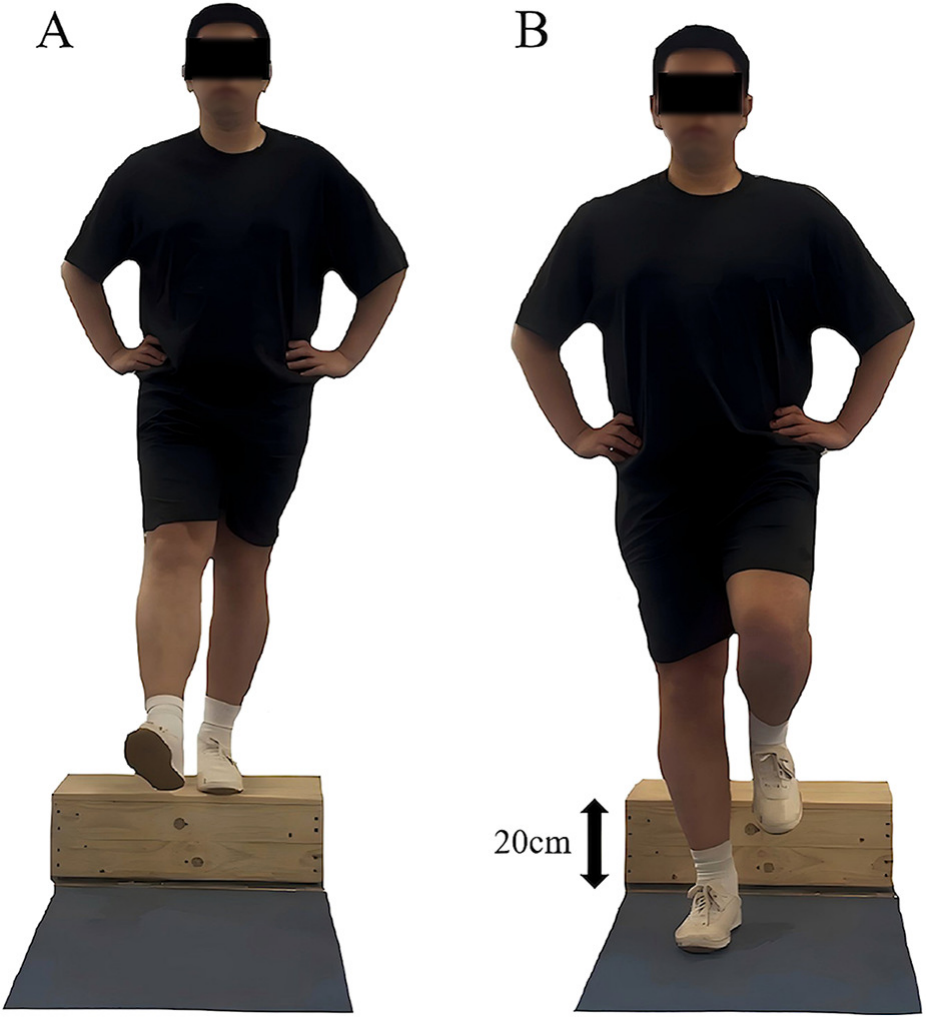
Illustration of the dynamic postural stability test. **(A)** Starting position. **(B)** Ending position. Right leg represents the affected side, while the left leg represents the unaffected side.

Participants performed a drop-landing task to assess dynamic postural stability (43). Standing on a 20 cm wooden platform in front of a force plate, they positioned feet shoulder-width apart, hands at their waist, and gaze fixed forward. Following instructions, participants stepped forward with their affected limb, dropped onto the force plate, and stabilized on the affected leg for 5 s (Figure 3) for three trials. A successful trial required landing without losing balance or corrective movements. Prior to the formal testing, participants completed three practice trials to become familiar with the procedure.

### 2.8 Data reduction

During static postural stability test, CoP_RMS was calculated from anteroposterior (AP) and mediolateral (ML) directions using CoP data sampled at 1,000 Hz. The raw data were filtered using a fourth-order low-pass Butterworth filter with a 12 Hz cutoff frequency (14). Filtered data were used to compute CoP_RMS (mm) for each participant using the following formulas (14):

(1) $$\mathrm{CoP}\_\mathrm{RM}S_{\mathrm{ap}}=\sqrt{\frac{\sum {\left(x_{i}-\bar{x}\right)}^{2}}{N-1}}$$

(2) $$\mathrm{CoP}\_\mathrm{RM}S_{\mathrm{ml}}=\sqrt{\frac{\sum {\left(y_{i}-\bar{y}\right)}^{2}}{N-1}}$$

where *x_i_* and *y_i_* represent CoP coordinates in AP and ML directions, while *x* bar and *y* bar denote their means. The denominator *N*−1 reflects sample-based calculation.

**Figure 4:**
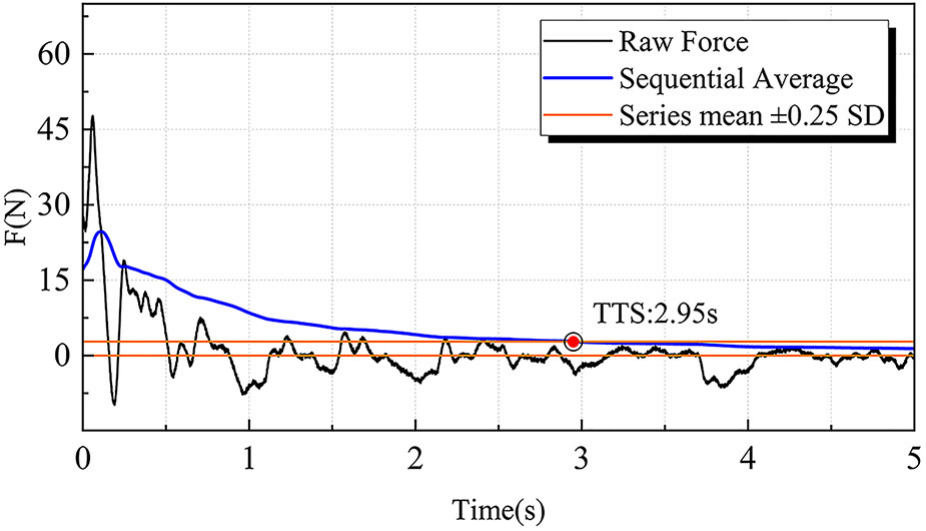
Illustration of the time to stabilization (TTS) calculation. The moment when the sequential average of ground reaction forces in the anteroposterior and mediolateral directions reaches and remains within the range of the series mean ± 0.25 SD is defined as the TTS. TTS, time to stabilization.

During dynamic postural stability test, ground reaction force (GRF) data were recorded at 1,000 Hz and filtered using a fourth-order low-pass Butterworth filter with a 12 Hz cutoff frequency (14). Filtered data from initial landing (GRF > 10 *N*) to 5 s post-landing were used to compute time to stabilization (TTS) through sequential average using the following formulas (44):

(3) $$\mathrm{Sequential}\;\mathrm{Average}\;\mathrm{TT}S_{\mathrm{ap}}(n)=\overset{1000}{\underset{n=1}{\sum }}\;\mathrm{Fx}/n$$

(4) $$\mathrm{Sequential}\;\mathrm{Average}\;\mathrm{TT}S_{\mathrm{ml}}(n)=\overset{1000}{\underset{n=1}{\sum }}\;\mathrm{Fy}/n$$

where Fx and Fy represent AP and ML GRF components. TTS was defined as the time from landing to when the sequential average of each component remained within ±25% of the standard deviation of the overall mean GRF for ≥1 s (45) (Figure 4).

### 2.9 Statistics analysis

Normality was confirmed using the Shapiro–Wilk test. A two-way mixed-design ANOVA evaluated main effects and interactions. Group (HD-tDCS + BBT vs. BBT) was specified as the between-subjects factor, and time (week0 vs. week7) as the within-subjects factor. Significant interactions were decomposed using Bonferroni-adjusted *post hoc* pairwise comparisons with correction for multiple testing. Effect sizes were reported as partial eta squared (*η*^2^*ₚ*: small = 0.01–0.06, moderate = 0.06–0.14, large > 0.14) for ANOVA results (46) and Cohen's *d* (trivial < 0.20, small = 0.21–0.50, medium = 0.51–0.80, large > 0.81) (47) for *post hoc* contrasts. Data are presented as mean ± standard deviation (SD). Significance was set at *p* < 0.05.

## 3 Results

**Figure 5:**
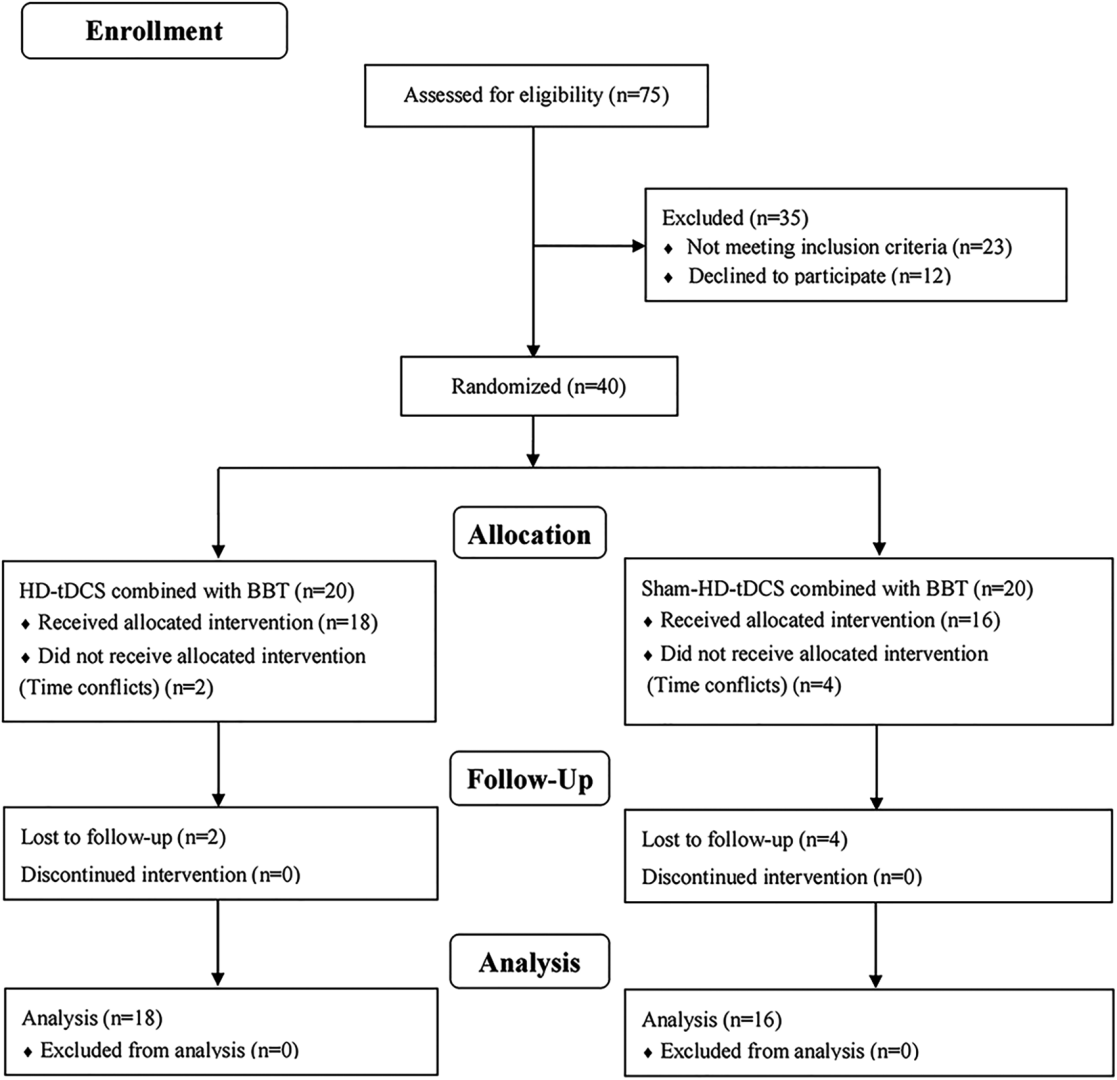
Participation flow chart. Final analysis included data from 34 participants. 41 participants were excluded from the original 75 recruited due to various reasons; HD-tDCS, high-definition transcranial direct current stimulation; BBT, Bosu ball training.

All dependent variables were normally distributed. Forty participants were randomly assigned to HD-tDCS + BBT (*n* = 20) or BBT (*n* = 20) interventions (Figure 5). Six withdrew due to scheduling conflicts, leaving 18 (HD-tDCS + BBT: 20.1 ± 1.3 years, 175.5 ± 8.0 cm, 72.4 ± 9.6 kg) and 16 (BBT: 21.0 ± 1.8 years, 173.3 ± 12.0 cm, 68.9 ± 11.5 kg) participants in each group. No between-group differences in age, height, or body mass existed (*p* > 0.05).

**Figure 6:**
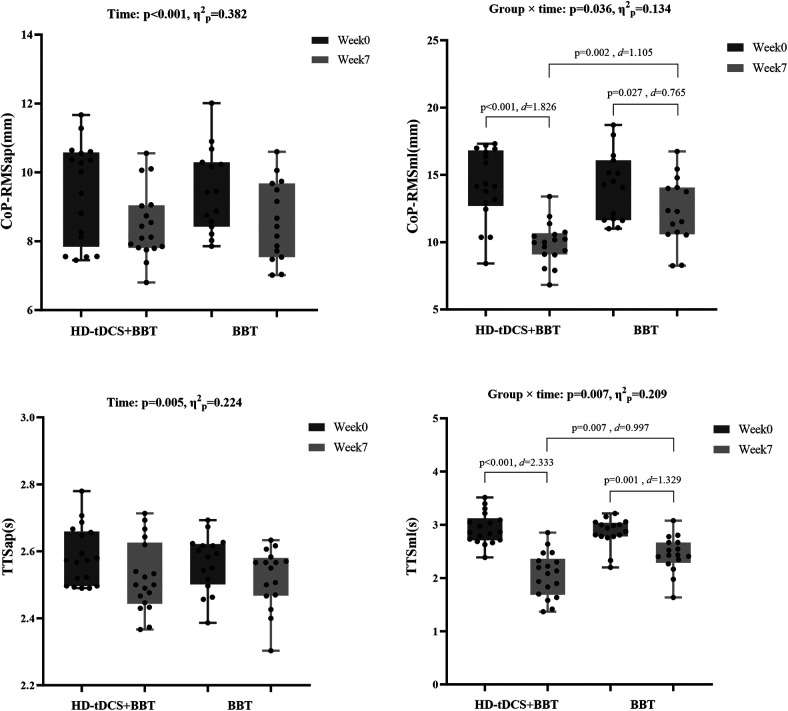
Static and dynamic postural stability before and after intervention. CoP_RMS, root mean square of the center of pressure; HD-tDCS, high-definition transcranial direct current stimulation; BBT, Bosu ball training; TTS, time to stabilization; ap, anterior-posterior direction; ml, medial-lateral direction.

Figure 6 revealed a significant group × time interaction for CoP_RMS_ml_ (*p* = 0.036, *η*^2^*ₚ* = 0.134). *Post hocs* showed both interventions reduced CoP_RMS_ml_ from week0 to week7 (HD-tDCS + BBT: *p* < 0.001, *d* = 1.826; BBT: *p* = 0.027, *d* = 0.765), with a greater reduction in the HD-tDCS + BBT intervention (*p* = 0.002, *d* = 1.105). CoP_RMS_ap_ exhibited a main effect of time (*p* < 0.001, *η*^2^*ₚ* = 0.382), with decreases after interventions.

Figure 6 showed a significant group × time interaction for TTS_ml_ (*p* = 0.007, *η*^2^*ₚ* = 0.209). *Post hocs* indicated reduced TTS_ml_ over time in both groups (HD-tDCS + BBT: *p* < 0.001, *d* = 2.333; BBT: *p* = 0.001, *d* = 1.329), with a larger decrease in the HD-tDCS + BBT intervention (*p* = 0.007, *d* = 0.997). TTS_ap_ demonstrated a main effect of time (*p* = 0.005, *η*^2^*ₚ* = 0.224), with reductions after interventions.

## 4 Discussion

This study investigated the effects of HD-tDCS combined with BBT on static and dynamic postural stability in individuals with CAI. Our results supported Hypotheses 1 and 2, demonstrating that both HD-tDCS + BBT and BBT alone significantly reduced CoP_RMS and TTS. And, HD-tDCS + BBT elicited greater improvements compared to BBT alone, suggesting enhanced efficacy of the combined intervention for postural stability.

### 4.1 Bosu ball training improved static and dynamic postural stability

This study demonstrates that both active and sham HD-tDCS, when combined with BBT, significantly improved static and dynamic postural stability in individuals with CAI in both AP and ML directions, underscoring the effectiveness of BBT as a rehabilitative intervention. These findings align with prior research: one study reported that unstable surface training enhances postural stability compared to stable surfaces by increasing neuromuscular demands and sensory integration (32), while another showed that such training elevates muscle activation and proprioceptive feedback to optimize joint stabilization (48). The observed improvements in postural stability following BBT in CAI may stem from its dual mechanisms of enhanced sensory input and neuromuscular adaptation. The compliant surface of the Bosu ball increases sensory stimulation by altering foot-support contact and pressure distribution, which amplifies proprioceptive input (49, 50). A meta-analysis confirmed that augmented sensory input significantly enhances postural stability in CAI populations (51), aligning with evidence of a strong correlation between proprioceptive acuity and postural control in this cohort (52). The inherent instability of the Bosu ball introduces controlled postural perturbations, promoting sensory reweighting—a CNS process that recalibrates reliance on visual, vestibular, and somatosensory inputs to compensate for instability (53). This adaptive mechanism synergizes with neuromuscular demands, as maintaining balance on an unstable surface requires dynamic adjustments to the center of gravity within functional limits (54, 55), thereby counteracting CAI-related deficits in lower limb strength and postural instability (56). Notably, a 4-week unstable surface training protocol improved both muscular strength and postural stability in CAI (48), supporting the results that compliant surfaces elevate neuromuscular demands, fostering enhanced muscle activation and force generation (57). Consequently, these neuromuscular adaptations contribute to improved postural stability in individuals with CAI.

### 4.2 Superior effects of combined HD-tDCS and Bosu ball training over Bosu ball training alone

Our findings demonstrate that HD-tDCS combined with BBT exhibited superior efficacy compared to BBT alone in enhancing static and dynamic postural stability among individuals with CAI, these results align with previous studies showing that HD-tDCS paired with foot-core exercises improves proprioception and static balance in healthy adults (36), and that targeting M1/S1 with HD-tDCS during short-foot exercises enhances proprioception and dynamic balance in CAI populations (42). Additionally, evidence that anodal tDCS over M1, when coupled with targeted muscular attention, augments motor cortex plasticity—evidenced by increased motor evoked potentials and reduced short-interval intracortical inhibition—further supports the synergistic effects of HD-tDCS and sensorimotor training on postural stability and motor learning (58, 59).

The superior efficacy of the combined intervention may be attributed to enhanced somatosensory integration, facilitated by HD-tDCS-induced neuromodulation of sensorimotor networks. Primary, HD-tDCS over S1 and M1 likely optimizes cortical excitability, improving sensory processing and motor output during BBT. This aligns with prior work demonstrating that tDCS enhances peripheral somatosensory acuity—evidenced by reduced vibration detection thresholds at the plantar surface and improved hallux sensitivity—thereby refining foot-ankle sensorimotor integration and postural control (60, 61). Such effects may stem from tDCS-mediated modulation of S1 excitability, which could synergize with proprioceptive training to enhance dynamic stability (62). Furthermore, tDCS applied over adjacent temporal-parietal regions has shown benefits for vestibulo-perceptual function (63), suggesting that stimulation effects may extend beyond targeted areas (M1/S1) to interconnected cortical networks involved in multisensory integration, collectively contributing to improved postural outcomes. Secondary, HD-tDCS may enhance postural stability via M1-mediated modulation of lower limb motor output. Individuals with CAI exhibit reduced M1 excitability projecting to the peroneus longus compared to controls (57, 64). Anodal tDCS increases M1 excitability by decreasing resting membrane potential in targeted regions, thereby augmenting corticospinal drive (34). This neuromodulation persists post-stimulation, reducing short-interval intracortical inhibition and enhancing voluntary muscle activation, which strengthens peroneal muscle contributions to postural control (59, 65).

### 4.3 HD-tDCS induced additional improvement of the postural stability in the ML direction

Our results indicate that the additional benefits of HD-tDCS occur specifically in the ML direction. This finding is partially supported by a previous study, which demonstrated that compared to a 4-week foot core training program, HD-tDCS improved passive kinesthesia thresholds for ankle inversion and eversion in healthy individuals, but had limited effects on ankle proprioception for plantarflexion and dorsiflexion (36). Neuromuscular control systems exhibit direction-dependent modulation in postural compensation responses. During perturbations in AP direction, postural stability is maintained through coordinated limb swing patterns and compensatory foot displacement (66). Conversely, perturbations in the ML direction may present greater neuromuscular challenges due to anatomical constraints in lateral limb repositioning (66). The stabilization response in the ML direction initiates with activation of the ankle eversion muscles to counteract inversion stresses (67, 68), a mechanism potentially compromised in individuals with CAI, thereby increasing the risk of sprain recurrence. This directional situation shows a higher correlation between ML stability deficits and fall risk compared to AP instability (69).

## 5 Limitations

There are several limitations to this study. Firstly, the study compared the effects of active vs. sham HD-tDCS combined with BBT on improving postural stability in individuals with CAI. However, the isolated effects of HD-tDCS remain undetermined. Despite this, our findings demonstrate that cortical stimulation significantly enhances the efficacy of BBT, establishing a clinically relevant physical therapeutic paradigm. Secondly, the study focused on the outcomes of a six-week intervention without evaluating long-term efficacy, which limits conclusions about the sustained effects of the intervention. Nevertheless, the study demonstrated clear improvements in postural stability, suggesting the potential value of prolonged intervention.

## 6 Conclusion

Both HD-tDCS + BBT and BBT alone significantly improves static and dynamic postural stability in individuals with CAI, while the combination of HD-tDCS and BBT is more effective than BBT alone, particularly in the ML direction. These findings highlight the potential of combining CNS interventions with peripheral therapies to improve postural instability for individuals with CAI.

## Data Availability

The raw data supporting the conclusions of this article will be made available by the authors, without undue reservation.
